# Outlines of Pathology and Practice of Medicine

**Published:** 1844-07

**Authors:** 


### REVIEWS.

---

Art. IV.—*Outlines of Pathology and Practice of Medicine.* By William Pulteney Alison, M.D., F.R.S., Fellow and late President of the Royal College of Physicians, Edinburgh; Professor of the Practice of Medicine in the University of Edinburgh, &c., &c. Philadelphia: Lea & Blanchard. 1844. pp. 424. 8vo.

Some months since we had the pleasure of introducing to the attention of our readers a work on the Principles of Medicine by Dr. Williams, and through the politeness of the publishers we are able to present an early account of the treatise of Dr. Alison on the same subject. These works, though professedly treating of the same general topics, and characterized each by uncommon excellence, yet differ so materially in their arrangement, and the style in which the views of their authors are carried out, that they may be read in immediate succession without any feeling of weariness from repetition. They do not cover the same ground, and are in no respect rivals, and hence neither may be expected to supersede the other. The work of Alison evinces greater discipline of mind, and, perhaps, would be styled the more finished treatise of the two; but that of Williams has more freshness about it, a greater leaning towards the discoveries of chemistry and the microscope, and on that account may be taken up with advantage at the point where the inquiry of Alison leaves off. The two works agree in insisting on the need of *principles in medicine*, concerning which Dr. Alison thus expresses himself: "notwithstanding all that has been said, and may be said, against medical speculation, I am fully convinced of the truth of the observation of Dr. Cullen, that 'at all times the practice of medicine has been and still is, with every person, founded more or less upon certain principles established by reasoning.'" Thoroughly persuaded of the truth of this observation, it is with sincere pleasure that we hail the appearance of every new treatise in which these principles are clearly and ably exhibited. Every such work is a benefaction for which all enlightened physicians will be ready to give thanks and honor to the author.

Such a work, we have said, is the one the title of which is prefixed to this article. The author of it is one of the most experienced of European physicians. He long held the chair of the Institutes in the school in which he now holds that of the Practice of Medicine, and thus he brought to the task an amount of training which, aided by a familiarity with disease at the bed-side, could not fail to impress upon his Outlines a character of the most substantial usefulness. The term is, perhaps, somewhat vague, but we cannot think of one which better expresses our idea of the distinguishing features of this work, than to say that it is eminently philosophical. It presents a clear outline of the philosophy of medicine, avoiding the extreme of speculation, on one hand, and on the other that mere jumble of cases which, whatever may be their value as a series of ascertained facts, cannot lay claims to being anything higher than a rational empiricism. The principles laid down in it have their foundation in facts which have been carefully observed, and the facts are so connected by principle as to give to them not the semblance merely but the reality of genuine science.

Having said thus much in commendation of the work, we proceed to quote such passages as we marked in a careful perusal of it from which we have just risen, by which, we feel very confident, our good opinion will be admitted by our readers to be fully sustained. Anything like a complete analysis of a work from which details have been so far excluded, it is quite plain, would be out of the question in the limits of one article. We shall, therefore, make up our notice chiefly of of such extracts as struck us while reading the volume as most likely to interest practitioners of medicine.

The work is divided into three parts, of which the first treats of cases of sudden and violent death; of diseases in general, their fatal terminations or spontaneous decline; of the remote causes of disease, and the means of their prevention; and of the action of the remedies in general, and the evidence of their efficacy. Part 2d treats of febrile diseases, embracing all inflammations; and part 3d considers chronic or non-febrile diseases in general, including those of the heart, lungs, stomach, organs of generation, and nervous system. "The object of Pathology," says the author, "is to describe and explain all the phenomena by which the diseased states of the living body differ from the healthy; and we call states of the living body diseased, in which there are such deviations from its natural condition as cause suffering or inconvenience, or endanger life." The subject being thus defined, he proceeds with a very scientific account of the causes leading to sudden death, which he declares to be such as *directly depress* or *suspend the vital action of the organs of circulation*, or else *obstruct the arterialization of the blood*, and therefore, according to principles known in physiology, *arrest the circulation at the lungs*." From which short quotations the reader will perceive, that the volume before us, while it is marked by a clearness of thought and precision of language most delightful to the student, is one which is not to be understood without study. We make a few extracts from part I.

The following paragraph bears favorably upon organic chemistry. Some might reject it as having a squinting towards the humoral pathology. It nevertheless conveys an instructive truth. It runs thus:

"In the case of certain local diseases, particularly of the liver and kidneys, a very peculiar mode of fatal termination is sometimes observed, in consequence of the local disease leading to retention of the natural excretions, and thereby affecting the system as a narcotic poison. And it is also certain, that death from any of the strictly febrile diseases, and from certain of the inflammations which we term specific, is owing, in part, sometimes almost entirely, to the influence of causes which affect peculiarly the fluids of the body, without altering the structure of any of its solid parts, and produce changes analogous in the most important respects to the agency of poisons on the animal economy."

In reference to the tendency of most diseases to a favorable termination, he has the following remarks:

"Another provision by which the injury done to individual parts of the body, both by inflammation and other diseases, is obviated in various cases, is the obstruction to the circulation through them, and the increased flow to, and increased development of, neighboring or corresponding parts, by which the place of the injured organ is supplied.

"The state of hypertrophy into which the heart and other muscular organs are brought, by the existence of any cause which obstructs the result of their natural action, is another provision by which the injurious effects of very common diseases are lessened or retarded. And we are by no means entitled to set aside the supposition of many pathologists, that the state which we call febrile reaction, is a provision by which the organs of circulation are excited to resist the noxious effects of causes which would otherwise irretrievably depress their action, in like manner as the state of hypertrophy is that, in which the heart permanently reacts against causes which would otherwise arrest the flow of blood."

Among the remote causes of disease he includes plethora as one. He says:

"Those whose knowledge of diseases is chiefly taken from examinations after death, as they generally find sufficient lesions of individual organs to explain the symtoms and event, see little of the evidence which establishes the importance of general and local plethora as a cause of disease; but those who are accustomed to trace the whole progress of individual cases, and observe the effects of remedies and regimen on them, have ample grounds for the belief that plethora, general and partial, is one of the most frequent, and often the most remediable, of the causes to which attacks, and still more frequently recurrences, of various local diseases, whether inflammatory, hæmorrhagic, or more chronic, may be traced."

In our next quotation the author takes a step very much in advance of those physiologists who deny that medicines ever reach the circulation. His speculation has at least ingenuity to recommend it. Speaking of the *modus operandi* of medicines, he remarks as follows:

"It is obvious that the great desideratum in therapeutics at present is, to establish the laws according to which the vital affinities of the minute particles of the blood and the living solids may be altered by the agency of remedies; and that when this is done, most of the remedies which we now describe, somewhat vaguely, as stimulating secretions, excretions, absorption, &c., or even as specifics, will be regarded as acting primarily on the blood, and on the vital changes which are continually going on in its substance, and which are essential to its healthy constitution."

The account given by our author of inflammation is as satisfactory as it could be made in the present state of our knowledge. Inflammation, he holds, depends upon *vital* and not mechanical causes. It is not a mere obstruction in the flow of the blood, as taught by some, nor does the relaxation of the vessels which attends it fully explain the phenomena. The author sums up in the following manner:

"We are thus led, by ascertained facts, and, as we believe, irresistible inferences from these, to the conclusion, that inflammation and its effects are inexplicable by any alteration of the contractile power of the living solids concerned in it; and necessarily imply an alteration of vital properties, by which the constitution of the blood, its relations to the surrounding textures, and its movement through them, are determined, but which are quite distinct from any contractions of living solids. That such living properties exist, that they effect the changes taking place at *insensible distances* among the particles of the blood, and that they are altered in inflammation, will hardly be denied by any pathologist. That they are capable of affecting the *visible* motion of the blood, will appear a rash assertion only to those who have not accustomed themselves to consider the evidence by which it is supported.

"From all these facts we think ourselves justified in inferring, that inflammation consists essentially in a local increase of a vital property of attraction existing among the particles of the blood, and between them and the surrounding textures, and with which other vital properties are connected, and simultaneously excited. That the proximate cause of inflammation, although affecting the constitution of the blood, does not reside in the blood only, but primarily in the agency on the blood of the solids through which it passes in the capillary vessels, appears clearly from the limitation of the disease, to a certain locality in the body, from the fact of its easy reproduction, for a long time, or for life, in the vessels which have once been the seat of it,—and from other facts to be mentioned as to inflammatory effusions. In the blood itself, the increased attraction seems to take place, both among the particles of the fibrin which, in union with the serum, constitute the liquor sanguinis in the living vessels, and in those particles of nearly similar matter, which constitute the bodies of the globules; the increased aggregation of both, and increasing number of the former, being characteristic of inflamed blood."

Concerning the use of pus Dr. Alison expresses the following opinion:

"Mr. Hunter puts the question, 'what is the use of the secretion of pus?' and confesses himself unable to answer it. We may approximate, at least, to an answer by observing, that all living action, without exception, is probably attended with diminution or loss of vitality in the acting particles, and thereby ultimately leads to the formation of fluids, which, if retained in the living body, act as poisons. The locally increased and altered living action of inflammation, when of a certain duration, and in a certain lower grade of intensity, or at a certain distance from the living influence of the existing solid, is subjected to the same law, and can only terminate favorably by the expulsion of the excretion which is thus formed. We shall afterwards see, that it is at all events a fact, that, whenever purulent matter is directly mixed with the blood, it acts on the system as a poison; and that its very rapid formation is characteristic of a specific form of inflammation, attended with depression of vital power."

In the section which treats of the modes of fatal termination of inflammatory diseases, we find the following instructive observations:

"Inflammation is sometimes quickly fatal, independently of any alteration in the texture of the inflamed part, simply by the gradual depression of the powers of the circulation which attends it, and which may be called a strictly sympathetic effect. The case of inflammation of the peritoneum is the best example of this danger. The effects of this inflammation, perceptible on dissection, bear no fixed proportion to the intensity or rapidity of the symptoms; and the disorganization which is found, effusion of serum, of lymph, or of pus, thickening of the membrane, even gangrene of the membrane (which is more rarely found), although they may explain torpor of the intestines, afford no explanation of the gradual but rapid depression and ultimate extinction of the heart's action, and consequent coldness of the surface, otherwise than by reference to the principle already explained, that any violent injury of the abdomen, by virtue probably of the intense and peculiar sensation it excites, acts as a powerful sedative on the heart.

"It is in this manner almost exclusively that inflammation of this part produces death, and it affords the best example of death beginning at the heart, and uncomplicated with simultaneous affection of the brain, which occurs in any disease."

A case strikingly illustrative of the truth of the above remarks occurred in our practice during the last autumn. A negro woman in excellent health, was brought to bed with her second child, and in four weeks was so well that she resumed her business as cook for a large family. The evening after commencing this work, she experienced some chilliness, which was followed by pain in the pelvic region, and a bad night. Next morning the pain had become severe, but the family not regarding the case as anything more than one of colic prescribed some simple remedies and it was suffered to take its course. The following night was one of much suffering, and very early the next morning the patient was discovered to be laboring under a severe chill. We saw her at 8 o'clock, at which time she appeared to be in a state of collapse—with cold surface and extremities, small, thready pulse; short, laborious breathing; complaining, when aroused, of pain low down in the abdomen. Efforts were made to excite reaction by wine, ammonia, sinapisms, hot applications and blisters, but the fatal symptoms advanced, and she died in an hour after we first saw her. A *post-mortem* examination was made in the afternoon, by which extensive inflammation of the peritoneum was revealed. The substance of the uterus and its appendages, as well as its peritoneal covering were inflamed. A pint of serum was found in the pelvis containing shreds of lymph. In this case death followed in less than forty hours the first symptoms of peritonitis. What might have been done by the proper treatment in the first few hours of the disease, it is impossible to say. According to our author:

"No proposition in medical science is more certain, and certainly no one is more practically important, than that which regards the power of large and repeated blood-letting to arrest the progress of inflammation in its early state, before any great amount of effusion has taken place,—and to cause many cases of it to terminate favorably by resolution, with such slight effusion only as is afterwards easily absorbed; which would otherwise have gone on to extensive and probably fatal disorganization of the different kinds above mentioned. Indeed, it has been stated, and probably with truth, in relation to healthy inflammation occurring in a sound constitution, and unattended with mechanical injury to the parts concerned, that "every constitution which is capable of having such inflammation excited in it, is capable also of bearing the evacuations, chiefly of blood, by which that inflammation may be subdued."*

This remark applies only to inflammations of a general

---

* Bateman.

character, or, as styled, "healthy inflammation," and is not true of those which are specific in their nature. Of the latter class Alison mentions three varieties—the *rheumatic*, the *gouty*, and the *syphilitic*. In regard to these he says:

"That inflammation may exist of a nature not to be subdued, even to be aggravated, by blood-letting or other evacuations, is quite certain from such experiments as those of Magendie as to the eye, and Gendrin as to the stomach; in which the kind of inflammation of the mucous membrane formerly mentioned was brought on by inanition, and could only be relieved by fuller nourishment, restoring the strength of the circulation, and probably restoring to the mucous membrane its natural protecting mucus; and that the kind of inflammation which is recognized in a patient affords very often a reasonable ground of objection to full blood-letting is sufficiently obvious when we attend to the known history of scrofulous, rheumatic, and gouty inflammation. In the first of these, it is true, that, on occasion of a recent inflammatory attack, when the symptoms approach most nearly to those of healthy inflammation, we have every reason to believe that blood-letting is often of the most essential importance, preventing aggravation of disease already existing, or arresting disease which would otherwise be established. But scrofulous inflammation is less under the influence of blood-letting than healthy inflammation; and scrofulous diseases occur chiefly in weakly persons, in those whose mode of life in early youth has been debilitating, and in those recently weakened by any considerable evacuations. Therefore, by full and repeated blood-letting in scrofulous cases, while we make little impression on the inflammation that exists, we incur a great risk of so far lowering the constitution as to make it more liable than previously to fresh attacks of inflammation, or to other scrofulous diseases, perhaps not inflammatory in their origin."

Of opium as an auxiliary to venesection in certain inflammatory diseases our author thus judiciously remarks:

"That opium is an important and valuable auxiliary to blood-letting in abdominal inflammation, is a principle which seems firmly established by the practice of various physicians in this country. It appears that its value (except as a palliative to uneasy feelings) is nearly confined to the inflammations of the intestines, enteritis, and dysentery, and that it is important in them, not to diminish the quantity of blood which should be drawn to subdue the inflammation, but to relieve those very oppressive sensations which seem to be the connecting link between the inflammation in the intestines and the actions of the heart, and by which these actions are so often rapidly and irretrievably depressed. Under the full use of opium, after bleeding in these diseases, these feelings are often relieved, vomiting allayed, sleep procured, (whether with or without sweating does not appear to be material), and the pulse is found to rise in strength; and if, as very generally happens in well-marked cases, the inflammatory symptoms recur, blood-letting may be repeated again and again, without symptoms of sinking. The disease is placed, as to its possible duration, and the effects of repeated blood-letting, more on a footing with inflammations of other parts, than it is when this auxiliary is omitted. Under this treatment, it may be stated with confidence, that the success of treatment in these inflammations, when attended by the peculiar depression of the circulation, and when the inflammation is of the healthy character, as distinguished from the erythematic and often epidemic peritonitis, is very considerably greater than when opium is withheld."

Bronchitis, of which our author observes, that in its sim plest and mildest form, it is the most frequent inflammatory disease produced by exposure to cold or wet, becomes in one shape an exceedingly formidable affection. In regard to that he remarks as follows:

"In such cases of violent bronchitis, the diagnosis from peripneumony, with or without pleurisy, becomes important, and is generally marked pretty distinctly by the absence of pain on either side, or of any stain of blood in the sputa, by the sonorous respiration, and the bronchial râles, heard chiefly on expiration, and pretty uniformly on both sides of the chest, and by the expansion of both sides on inspiration, and the sound on percussion in both, continuing natural. The diagnosis is remarkably aided, likewise, by the frequent exacerbations and remissions of the dyspnœa, the former of which, in the cases where there is most of the spasm, take place chiefly in the morning after sleep,—in those where inflammation predominates, chiefly in the evenings,—the remissions being generally marked by free expectoration."

In the next quotation, which we shall introduce from the same chapter, we have an explanation of that shortness of breath with which many old persons are troubled. The extract in a pathological point of view is highly interesting.

"The repeated recurrence of the disease, especially with asthmatic paroxysms, leads to the state called emphysema pulmonum, in which the septa of many of the minute air-cells are broken down, and they coalesce into large irregular cavities,—air escapes into the cellular substance immediately beneath the pleura, and sometimes forms bullæ on its surface,—and the whole lung becomes more voluminous, of lower specific gravity, and, by reason of the enlargement of the cells, approximates to the structure of the lungs of reptiles; and is rendered unfit for the rapidity of mutual action of the air and blood which is requisite in warm-blooded animals, at least under any excitement of the circulation. This state, therefore, necessarily implies habitual shortness of breath; and is very often indicated, as an accompaniment and result of habitual bronchitis and asthma, by the unnaturally arched and very resonant chest, with faintness of the natural respiratory murmur, and constant admixture of bronchial râles, chiefly in expiration. A certain degree of this change of the lungs, as Dr. Lombard has shown, takes place very frequently, and apparently from slight causes, in advanced life, when the texture of the lungs, particularly towards their external surface, becomes comparatively bloodless and brittle; but in younger persons it probably takes place only as a consequence of the bronchial inflammations connected with spasms,—*i. e.* asthma and hooping-cough,—and in such subjects no doubt admits of spontaneous cure, when recurrence of the inflammation is prevented."

Having urged the necessity of free depletion in peripneumony, the blood-letting to be large and continued until the breathing is distinctly relieved, and to be repeated sometimes four or six times, or even oftener, he goes on to remark:

"In many cases in the latter stages, when the active inflammation has subsided, the expectoration is promoted, and the strength and general feelings of the patient are benefited by the use of small quantities of stimuli, particularly wine and the preparation of ammonia; and in a few cases, neglected in the commencement, and where the symptoms of the advanced stage (frequent and soft pulse, dyspnœa, general mucous râles, and sputa deeply and darkly stained) are urgent, recoveries take place under the use of such stimulating remedies exclusively,—the inflammation having subsided spontaneously without extending over much of the lung."

In this connexion, the author alludes to two peculiar forms of pneumonia which from the nature of the case are generally fatal. The first, which is characterized by partial suppuration in the lungs, is to be apprehended, "when symptoms of peripneumony supervene on those of inflamed veins, or after small-pox, or more especially after the amputation of a suppurating surface, when it may be concluded that purulent matter is circulating in large quantities in the blood." The second is observed in cases where the pneumonic inflammation is complicated with some disease producing a feeble state of the circulation, and is denoted with certainty by the smell of the breath. Under the cautious use of stimulants Dr. A. thinks recoveries have taken place from the advanced stage of affections of both these kinds, but in such cases there is reason to believe the disease was more partial than usual.

It is not now so much the fashion as it was a few years since, to refer diseases of every sort to inflammation or congestion of the liver; but doubtless derangement of that organ is the *fons et origo* of many of the maladies which pass under the term bilious. Our author refers in the subjoined passage to an obscure inflammation of the substance of that organ:

"In such cases there are often various dyspeptic symptoms attending the disease of the liver; sometimes much vomiting, in other cases none,—sometimes complete anorexia, in other cases a morbidly keen appetite; often dryness of the tongue, and a very peculiar fetor of the breath; and usually either a defect or a morbid quality of bile in the stools; frequently there is discharge of blood by stool,—occasionally by vomiting; but in some instances we can hardly point out any other attendants than gradually increasing debility and emaciation, usually with a sallow complexion."

Under the same head he has the following observations:

"Enlargements of the spleen frequently coexist with those of the liver, particularly in those who have suffered with intermittent fevers; or exist alone, sometimes for a great length of time. They are often connected with repeated fits of hæmatemesis. In these cases, the most important practical observation is, that preparations of mercury are very apt to effect the system violently and injuriously; and that blisters, and frequently repeated laxatives, combined with bitters and preparations of iron, have seemed the most useful remedies."

It has already been mentioned that the author considers the inflammation of rheumatism as specific. In accordance with that view of the disease, he condemns active depleting remedies. His language is:

"The acute rheumatism cannot, probably, be much shortened in its duration by antiphlogistic remedies, and if it were so shortened in external parts, we have good reason to think that the risk of affection of the heart would be greatly increased. For although the translation to the heart has been observed to take place under all modes of treatment of the disease, yet it has been seen to follow large bleeding, and immediately consequent recession of the inflammation on the extremities, often enough and quickly enough to justify much apprehension of such a result, when the quantity of blood taken is so great as to produce a marked and immediate effect on the heart's action."

In chronic rheumatism of all the numerous remedies that have been used, the hydriodate of potass is the one most in vogue at the present time. We are acquainted with a gentlemen in whose case the medicine acts like a charm. We knew another gentleman, some years ago, who derived pretty certain relief from the use of the blue pill.

We will conclude this notice by a few extracts from part III. of this admirable work. This treats of *chronic or non-febrile diseases.* In these appear the following kinds of morbid structure; namely, *hypertrophy*, *atrophy*, *softening*, *hardening*, and various morbid growths: these are examples of *structu-*

*ral* disease. The *functional* diseases belonging to this class are numerous, and need not be recited. Among the causes leading to these complaints, our author regards a morbid change in the constitution of the blood, which may be brought about by the following agencies:

"1. By improper aliments, either defective in quality, or excessive, or unfitted for due assimilation.

"2. By truly poisonous matters (*e. g.* alcohol) introduced from without with the aliments, or by the lungs.

"3. By poisonous matters formed and retained in the body itself, though destined for excretion.

"4. By a defective or altered condition of those secretions which are immediately concerned in the assimilation of food.

"5. By a defective or altered state of other vital actions requisite for the assimilation of aliments, particularly of respiration, as affected by the quality of the air breathed, and of circulation, as excited by the natural stimulus of exercise.

"6. By other causes of excessive or deficient quantity of blood in individual parts, besides the nature or due preparation of the ingesta, *e. g.* such causes of local plethora as are stated above (p. 14), or such causes of locally defective circulation, as previously diseased or obstructed arteries.

"7. By causes, not yet understood, acting in individual parts of the body, and altering the constitution of the blood there in the first instance, which alteration is subsequently extended; as in inflammation, in tubercles, cancer, &c.

"8. By causes not yet understood, but which affect the vital properties of the whole blood, as is seen in cases of purpura or the hæmorrhagic diathesis.

"In all these cases it is a vital, not a simply chemical action, which is originally in fault, although in several a chemical change in the constitution of the blood, or of some of the secretions, results from, and indicates that altered vital action."

To the above causes he adds the following:

"1. In childhood there is often a deficiency of exercise, of mental excitement, and of the tonic influence of frequent changes of temperature.

"2. In youth there is often over-excitement of the nervous system by mental influences, combined with deficient exercise.

"3. Next, there is often intemperance of various kinds, affecting variously the function of digestion, the circulation, and the nervous system.

"4. In many there are too sedentary occupations, or only occasional and irregular muscular exertion, such as may excite disease rather than contribute to health.

"5. In many there is, as life advances, want of mental excitement and interest, along with want of exercise, especially in women.

"6. In others there is excessive exertion, mentally and bodily, deficiency of sleep, and mental anxiety.

"7. In many there is, independently of intemperance, repletion, and plethora from excess of food, and sometimes of sleep, and defect of exercise."

Dyspepsia has been styled the *Proteus of diseases*, assuming almost every shape. Instructive illustrations of this are given by our author in the annexed passage:

"All these forms of dyspepsia, although partly owing to known causes, suffer exacerbations for which we can assign no reason; and again, although often tedious, have the tendency to occasional spontaneous abatement. When unusually urgent, we suspect that they are connected with the subacute inflammation of the mucous membrane formerly considered; and when they last long, and are attended with much emaciation and weakness, we apprehend that they are connected with organic disease, either of the stomach itself, or of the viscera which adjoin it, or are connected in action with it in the modes formerly noticed.

"Even in youth, especially in young women, it is not uncommon to find the symptoms of dyspepsia—perhaps but slight—connected with ulcers on the mucous membrane of the stomach, which are preceded or attended with so little of the symptoms of inflammation, that they may be regarded as a form of chronic organic disease; and these may be suddenly fatal, either by erosion of a blood-vessel, and hæmorrhage, or (more frequently) by perforation of the coats, escape of the contents of the stomach, and rapid peritonitis.

"The other cases of organic disease of the stomach are most generally seated either at the cardia, or near the pylorus. In the former case, we have the pain and difficulty felt on the morsel of food passing into the stomach, and after a time very generally partial *regurgitation* of food. In the latter case, we have the accession of pain at regular times—generally from two to three hours after food is taken, followed and relieved by the periodical vomiting; the matter vomited often becoming ultimately of the dark brown or blackish color. In the case, not uncommon, where the stomach becomes greatly distended and enlarged, the vomiting is not so frequent or regular, but is occasionally to an enormous extent. In both cases, but especially in the latter, we may have, after a time, a small firm tumor perceptible to the finger, and somewhat tender on pressure, at the seat of the disease; which, however, is sometimes considerably moved from its natural position. Such diseases of the stomach are generally attended with obstinate costiveness."

But, if we went on quoting all the passages in this book by which our attention was particularly struck, we should hardly stop until our article had grown to the size of the volume itself. We have already made it as long, perhaps, as an article made up so much of extracts ought to be, and shall therefore take our leave of the work by again recommending it most heartily to the profession. Y.

